# Examining changes in vascular function, arterial stiffness and systemic inflammation during hospitalization and recovery from an acute exacerbation of chronic obstructive pulmonary disease

**DOI:** 10.1038/s41598-023-39001-z

**Published:** 2023-07-28

**Authors:** Desi P. Fuhr, Andrew R. Brotto, Brian H. Rowe, Mohit Bhutani, Rhonda J. Rosychuk, Michael K. Stickland

**Affiliations:** 1 Division of Pulmonary Medicine, Faculty of Medicine and Dentistry, University of Alberta, 3-135 Clinical Sciences Building, Edmonton, AB T6G 2J3 Canada; 2 Faculty of Kinesiology, Sport, and Recreation, University of Alberta, Edmonton, AB Canada; 3 Department of Emergency Medicine, Faculty of Medicine and Dentistry, and School of Public Health, University of Alberta, Edmonton, AB Canada; 4 Department of Pediatrics, Faculty of Medicine and Dentistry, University of Alberta, Edmonton, AB Canada; 5 G.F. MacDonald Centre for Lung Health, Covenant Health, Edmonton, AB Canada

**Keywords:** Vascular diseases, Vascular diseases, Outcomes research

## Abstract

An acute exacerbation of COPD (AECOPD) is associated with increased risk of cardiovascular (CV) events. The elevated risk during an AECOPD may be related to changes in vascular function, arterial stiffness, and systemic inflammation; the time course of these measures and their corresponding recovery are poorly understood. Further, physical activity is reduced during an AECOPD, and physical activity may influence the cardiovascular responses to an AECOPD. The purpose of the study was to examine the acute impact of an AECOPD requiring hospitalization on vascular function, arterial stiffness, and systemic inflammation and examine whether physical activity modulates these variables during recovery. Patients hospitalized for an AECOPD were prospectively recruited and compared to control patients with stable COPD. Vascular function, arterial stiffness, and systemic inflammation (CRP, IL-6) were measured at hospital admission, hospital discharge and within 14 days of discharge. Physical activity was electronically tracked daily while in hospital and for 7 days following discharge using a Fitbit. One hundred and twenty-one patients with an AECOPD requiring hospitalization and 33 control patients with stable COPD were enrolled in the study. Vascular function was significantly lower, and systemic inflammation higher at hospital admission in patients with an AECOPD compared to stable COPD. Significant improvements in vascular function and inflammation were observed within 14 days of hospital discharge; however, vascular function remained lower than stable COPD. Physical activity was low at admission and increased following discharge; however, physical activity was unrelated to measures of vascular function or inflammation at any time point. An AECOPD requiring hospitalization is associated with impaired vascular function that persists during recovery. These findings provide a mechanistic link to help explain the enduring increase in CV risk and mortality following a severe AECOPD event.

**Clinical trial registration**: ClinicalTrials.gov #NCT01949727; Registered: 09/20/2013.

## Introduction

Patients who experience an acute exacerbation of COPD (AECOPD) often seek medical assessment in emergency departments (ED) and many require hospital admission. Moreover, these patients experience a four-fold increase in cardiovascular (CV) events with an AECOPD, and a greater than two-fold increase in myocardial infarction risk within the first 5 days following an exacerbation^1^. The potential mechanism(s) for the increased CV and mortality risk during a severe AECOPD are unclear and requires further investigation.

Arterial stiffness and vascular function are both independent predictors of CV risk in healthy populations^2–7^ even when adjusted for traditional risk factors of CV disease such as age, sex, systolic and diastolic blood pressure, diabetes mellitus, total cholesterol, and smoking status^6^. In patients with stable COPD, arterial stiffness is elevated compared to age-matched controls^2–5^ and arterial stiffness increases during an AECOPD^8^. Vascular dysfunction, as evaluated by flow-mediated dilatation (FMD), is widely regarded as an early manifestation of CV disease^9^, and is impaired in patients with stable COPD^10^. An acute increase in inflammation appears to impair vascular function^11^, and a recent small study demonstrated that vascular function was lower during a severe AECOPD relative to healthy non-smokers^12^.

An AECOPD has a detrimental affect on multiple facets of everyday life including sleep, nutrition and physical activity^13,14^. Physical inactivity, an independent risk factor of CV disease^2,3,5^, is common in patients with stable COPD^15–18^ and physical activity is reduced even further during an AECOPD^19^. Patients hospitalized with an AECOPD undergo extended periods of bedrest. Prolonged bedrest in healthy participants has been shown to reduce CV function^20,21^ and increase inflammation^22,23^, while regular mobilization during prolonged bedrest has been shown to prevent the rise in inflammation^22^. The combined inflammatory response from a severe AECOPD requiring hospitalization as well as the hospitalization-associated bedrest may have synergistic effects and potentiate CV risk^22^ secondary to impairments in vascular function and arterial stiffness.

Several studies have examined connections between physical activity, arterial stiffness and systemic inflammation^24–29^; however, to our knowledge, no study has elucidated the pattern of inflammation during a severe AECOPD requiring hospitalization, and subsequently how physical activity may modulate inflammation, arterial stiffness, and vascular function. Thus, the purpose of the present study was to (1) examine the impact and time course of a severe AECOPD requiring hospitalization on vascular function, arterial stiffness, and systemic inflammation, and (2) examine whether physical activity was associated with vascular function, arterial stiffness, and systemic inflammation during an AECOPD.

## Methods

### Study design

The present study was a single-centre observational cohort study (ClinicalTrials.gov #NCT01949727) approved by the University of Alberta Health Research Ethics Board (Biomedical Panel, Pro00038838). Participants provided written informed consent prior to any research procedures and all methods were carried out in accordance with relevant guidelines and regulations. This study was performed in line with the principles of the Declaration of Helsinki.

### Measurement assessments

For each AECOPD inpatient, data were obtained over three periods; (1) within 24 h of admission to hospital (admission); (2) 24 h prior to discharge from hospital (discharge); and (3) within 14 days following discharge from hospital (14 Day Follow-up). Each testing day consisted of an assessment of vascular function, arterial stiffness, blood biomarkers and health related quality of life. Measurements were obtained at a consistent time to minimize diurnal variation and participants refrained from smoking a minimum of 12 h prior to assessments. Physical activity was assessed daily while in hospital and for a 7-day period following discharge. Control patients with stable COPD were assessed for primary and secondary outcomes at a single time point.

### Participants

**Figure 1:**
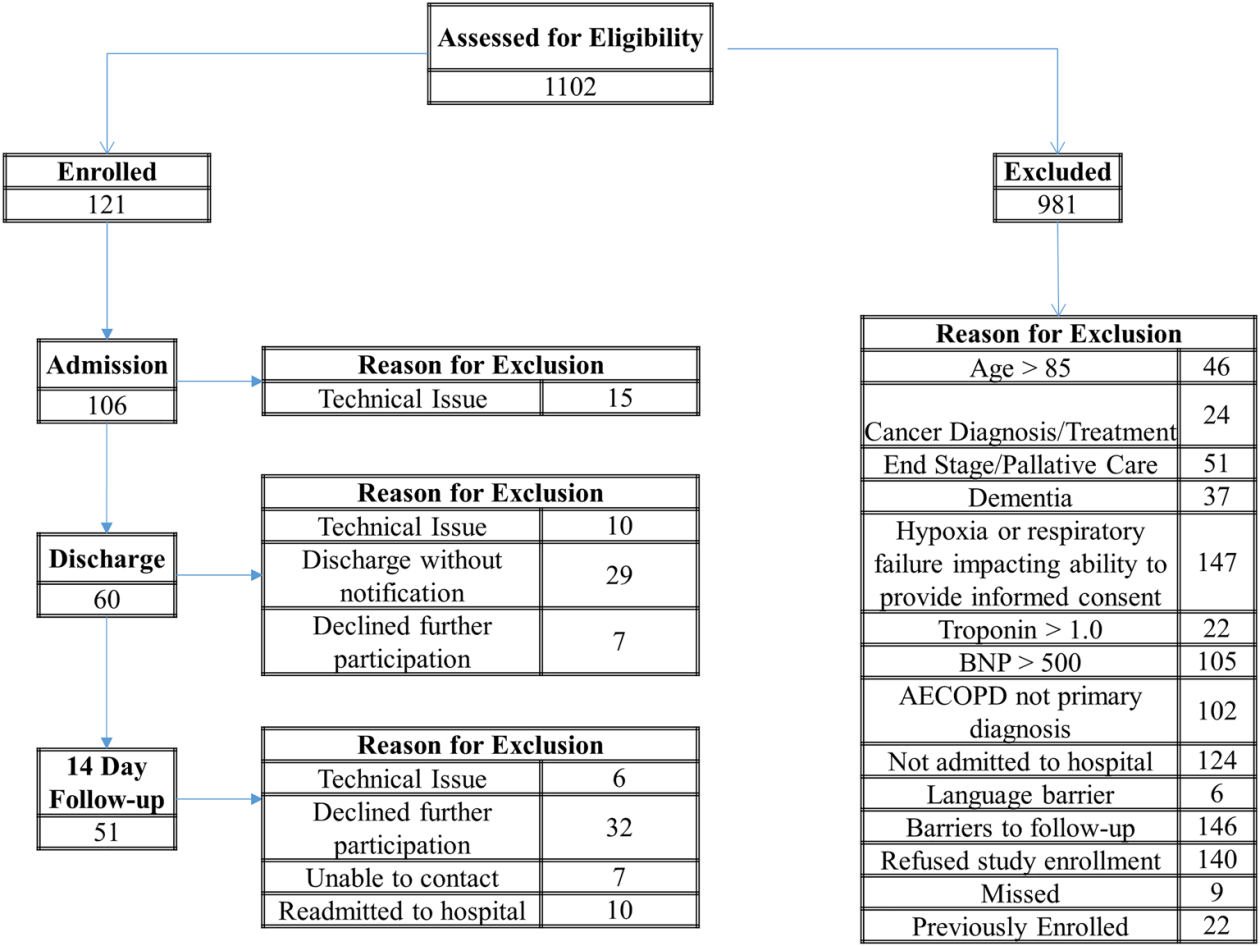
Patient eligibility, inclusion and exclusion. Trop: troponin; BNP: brain natriuretic peptide.

Adult patients (between 40–85 years of age) were enrolled if the primary diagnosis was an AECOPD and they were admitted to the University of Alberta Hospital (UAH) in Edmonton, AB (AECOPD inpatient). The UAH is a quaternary care, referral, academic teaching hospital with a large catchment area. Patients with respiratory failure impacting consent, conventional troponin-I (TnI) > 1.0 ng/ml, brain natriuretic peptide (BNP) > 500 pg/ml, or a diagnosis of cancer, dementia, palliative and/or end-stage disease were excluded (Fig. 1). A consort diagram indicating the number of patients assessed at Admission, Discharge and 14 Day Follow-up is available in Fig. 1. Patients that were not assessed at discharge were still contacted for the 14 day follow-up assessment. To confirm COPD, in-hospital spirometry (two flow-volume loops) was performed once on each patient with an AECOPD when they were sufficiently stable (typically within 2–3 days following admission) to provide reproducible maximal flow-volume loops.

For comparison, control patients with stable COPD were recruited from the community. All participants had COPD as defined by the American Thoracic Society criteria of irreversible post-bronchodilator airflow obstruction (i.e. FEV_1_/FVC below the lower limit of normal predicted from height, age, sex)^30^ and smoking history (> 10 pack-years). Stable COPD controls were classified as Gold Stage 1 and 2 or Gold Stage 3 and 4, had no history of an AECOPD within the previous 6 months, did not have cancer or dementia, and were not receiving end-stage care.

### Biomarkers

Clinical blood biomarkers of heart failure and cardiovascular disease (BNP and TnI)) were assessed while in the ED prior to hospital admission.

### Arterial stiffness

Following instrumentation, participants rested for at least 10 min in the supine position. Blood pressure and heart rate were recorded to confirm that the participant was stable before resting carotid-radial pulse wave data were collected. Pulse waveforms were simultaneously acquired by non-invasive pressure sensors (Complior, Alam Medical, Vincennes, France) from the carotid and radial arteries and used to calculate pulse wave velocity (PWV). Pulse wave velocity was calculated as *PWV* = *D∙Δt*^−*1*^, where *D* was the distance (m) between sites and *Δt* was the time difference (s) between pulse waves using the foot-to-foot method^31^. Distance between the recording sites at the carotid and radial arteries was measured on the surface of the body using a tape measure. Mean PWV was calculated as the average of at least five consecutive beats in order to cover a full respiratory cycle^31^.

### Vascular function

Vascular function was then assessed as outlined by Faizi et al.^32^ using the Endo-PAT^®^ (Endo-PAT^®^, Itamar Medical Ltd., Caesarea, Israel), which examines the vasodilatory response (measured at the finger) to 5 min of circulatory occlusion. Similar to other flow-mediated techniques, the vascular response assessed by the Endo-PAT^®^ has been shown to be related to coronary endothelial dysfunction^33^. Forearm occlusion was chosen to allow for consistency as the majority of patients had at least one intravenous site in the antecubital space which might be moved during hospitalization. Pulse amplitude response to hyperemia (RHI) was automatically calculated using the Endo-PAT^®^ proprietary algorithm^34,35^. Previous research has shown an RHI score of < 1.67 represents endothelial dysfunction^33,35^.

### Systemic inflammation

A venous puncture was performed by an experienced phlebotomist after arterial stiffness and RHI data were obtained. Blood samples were collected in plain vacutainer tubes and centrifuged for 10 min at 1200 rpm; serum was aliquoted (100 µl) into 0.6 mL cryovials. Samples were then stored at − 80 °C until analyzed. Serum analysis of human C-reactive protein (CRP) and interlukin-6 (IL-6) was performed using commercial ELISA assays (ELISA Human CRP assay and ELISA Human IL-6 assay R&D Systems, Minneapolis, MN), consistent with previous work^36,37^. CRP has previously been shown to be significantly related to flow mediated dilation^10^.

### Physical activity

Physical activity monitors (Fitbit, Fitbit Inc, San Francisco, CA) were worn by AECOPD inpatients at admission and at 14 day follow-up to assess number of steps taken during their hospital stay and while in the community^38^. The physical activity monitors selected for the study have been validated in patients with COPD both in the community and in a hospital setting^39,40^. Minute by minute data were extracted using the Fitbit developer application programming interface (Fitbit, Fitbit Inc, San Francisco, CA). Admission and discharge data represent the daily step count collected over that day for a patient, and 14 day follow-up represents the average step count measured over 7 days. Step counts were averaged over seven days in the control patients with stable COPD.

### Health related quality of life: CAT and mMRC

The impact of the AECOPD on the patient’s quality of life was evaluated over time using the COPD Assessment Test (CAT) and scores were totaled out of 40^41^. The modified Medical Research Council dyspnea scale (mMRC) questionnaire was used to assess perceived breathlessness^41^.

### Statistical analysis

Data are presented as mean with standard deviation (SD) or median with interquartile range (IQR) for continuous variables and as counts and proportions for dichotomous variables. Differences in baseline clinical characteristics, including smoking history and lung function, were evaluated using a one-way analysis of variance (ANOVA) followed by a Bonferroni-adjustment for multiple comparisons where statistical significance (p < 0.05) was found for continuous variables. A chi-square (χ^2^) test for was used for categorical variables.

Change in vascular function, arterial stiffness and systemic inflammation (primary outcomes) were analyzed using mixed effects linear regression models. The model had a fixed effect for timing discharge and 14 day follow-up, with admission as the reference level. The model allowed for easy reporting of the relevant comparisons of interest and had a random effect for subjects to capture within-subject dependence. Separate models also adjusted for the following covariates one at a time: age, sex, BMI, FEV_1_% predicted, mean arterial blood pressure (MAP), systolic blood pressure, diastolic blood pressure, length of stay (LOS) and estimated arterial O_2_ saturation (S_P_O_2_). Bonferroni correction was applied to the p-values to account for the three contrasts and a p-value of 0.05 was selected to indicate statistical significance. A Kruskal–Wallis test was used to determine statistical significance in step count between admission, discharge and 14 day follow up followed by a Bonferroni adjustment for multiple comparisons where statistical significance was set at p < 0.05.

In addition, Pearson correlations were used to determine if a relationship was present between primary outcomes and step count and partial correlations were used to determine the relationship between primary outcomes while controlling for step count.

All statistical analyses were performed in SPSS Statistical software version 26.0.0.0 IBM Corp™, Armonk, NY, USA, and Studio Team 2020, RStudio: Integrated Development for R. RStudio, PBC, Boston, MA, USA.

### Sample size

A convenience sample of COPD patients was recruited in an attempt to provide sufficient data for multiple regression analysis. Preliminary analysis indicated 100 COPD patients would provide reasonable confidence intervals on important variables and outcomes.

## Results

The study screened 1102 patients who presented at the ED for an AECOPD, and 121 patients were recruited prior to admission to hospital (Fig. 1, CONSORT diagram). Out of these, 111 were included in the analysis (AECOPD Inpatients). Baseline patient characteristics are outlined in Table 1. The groups were well matched for age, height, weight and smoking history. Patients with an AECOPD had similar airflow limitation (FEV_1_% predicted = 50 vs. 38), more likely to be on supplemental oxygen (49 vs. 23%), and had higher levels of perceived breathlessness as compared to stable patients with GOLD 3 and 4 COPD (see Table 1).

**Table 1:** Participant characteristics.

	Stable COPD controls GOLD 1 & 2	Stable COPD controls GOLD 3 & 4	AECOPD inpatients
n	11	22	111
Males/females, n	6/5	16/6	51/60
Age (years)	72 (9)	64 (9)*	69 (10)
Height (cm)	164 (10)	171 (9)	167 (11)
Mass (kg)	77 (20)	86 (23)	78 (24)
BMI (kg/m^2^)	28 (8)	29 (8)	28 (9)
Smoking history (pack years; median (IQR))	40 (32)	49 (15)	41 (25)
Length of hospitalization (days)	–	–	4.7 (3.1)
O_2_ supplementation (%)	0	23	49*
Anitbiotics (%)			79
Systemic corticosteroids (%)			82
Pulmonary function (% predicted)			
FEV_1_	87 (13)	38 (12)*	50 (22)*
FVC	97 (13)	72 (18)*	76 (26)*
FEV_1_/FVC %	86 (9)	53 (15)*	67 (24)*^¥^
Troponin (ng/ml)	–	–	0.1 (0.1)
Brain natriuretic peptide (pg/ml; median (IQR))	–	–	84 (137)
mMRC	1.2 (0.9)	2.4 (1.1)*	2.7 (1.2)*
COPD assessment tool	12 (8)	17 (7)	27 (8)*^¥^

Values are mean (SD) unless otherwise indicated.

Antibiotics and Systemic Corticosteroids prescribed during AECOPD.

*FEV*_*1*_ forced expiratory volume in 1 s, *FVC* forced vital capacity, *mMRC* modified Medical Research Council Questionnaire.

Summaries are based on completed data.

*p < 0.05 vs. Stable COPD control Gold 1 & 2.

^¥^p < 0.05 vs. Stable COPD control Gold 3 & 4.

The length of hospitalization for patients with an AECOPD ranged from 1 to 19 days with a median of 4 (IQR = 1, 7). There were no between-group differences in age, BMI, airway obstruction, smoking history or perceived dyspnea between inpatients with AECOPD and control patients with stable COPD (GOLD 3 and 4, Table 1). Health related QoL was significantly decreased at admission compared to control patients (Table 1) and improved significantly at 14-day follow up (see Table 2).

**Table 2:** Clinical characteristics of patients with an AECOPD from admission, discharge and 14 day follow-up (mean (SD)).

	Admission	Discharge	14 day follow up
Heart rate (bpm)	88 (17)	82 (13)*	80 (10)*
Systolic blood pressure (mmHg)	131 (24)	121 (17)*	122 (16)*
Diastolic blood pressure (mmHg)	76 (16)	70 (11)*	73 (9)
Mean arterial blood pressure (mmHg)	94 (15)	87 (11)*	89 (10)
Pulse oximetry (S_p_O_2_)	92 (5)	93 (3)	94 (3)
IL-6 (pg/ml)	7.2 (12.9)	9.9 (15.1)	8.6 (7.0)
mMRC	2.8 (1.2)	2.4 (1.0)	2.0 (1.0)*
COPD assessment tool	27 (8)	20 (7)*	18 (7)*
Step count (steps/day; median (IQR))	830 (1051)	964 (1844)	1740 (2830)*^¥^

Values are mean (SD).

Summaries are based on completed data.

*p < 0.05 vs. Admission.

^¥^p < 0.05 vs. Discharge.

### Vascular function, arterial stiffness, systemic inflammation

**Figure 2:**
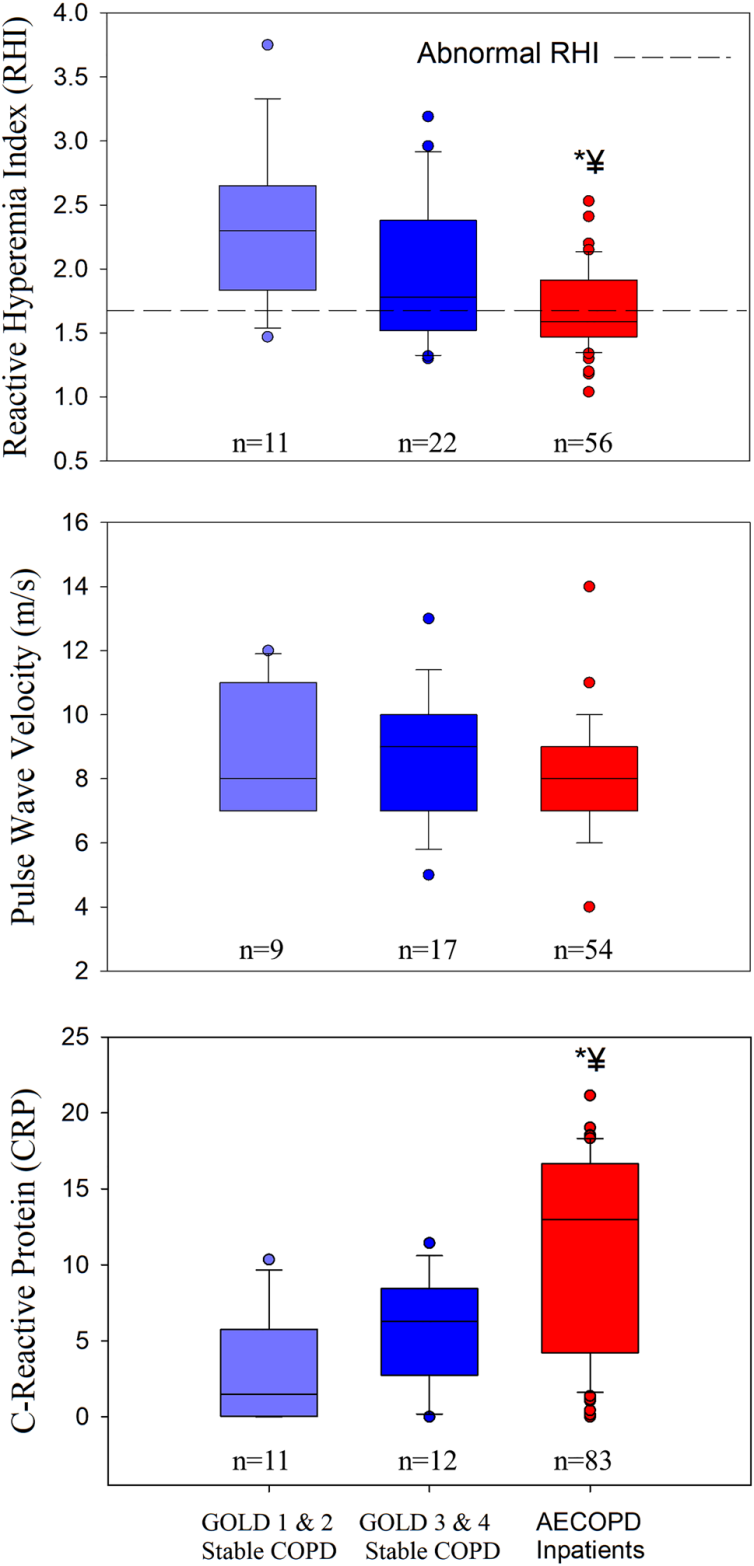
Vascular function, pulse wave velocity and systemic inflammation at admission. *p < 0.05 vs. GOLD 1 and 2 Stable COPD controls; ^¥^p < 0.05 vs. GOLD 3 and 4 Stable COPD control.

**Figure 3:**
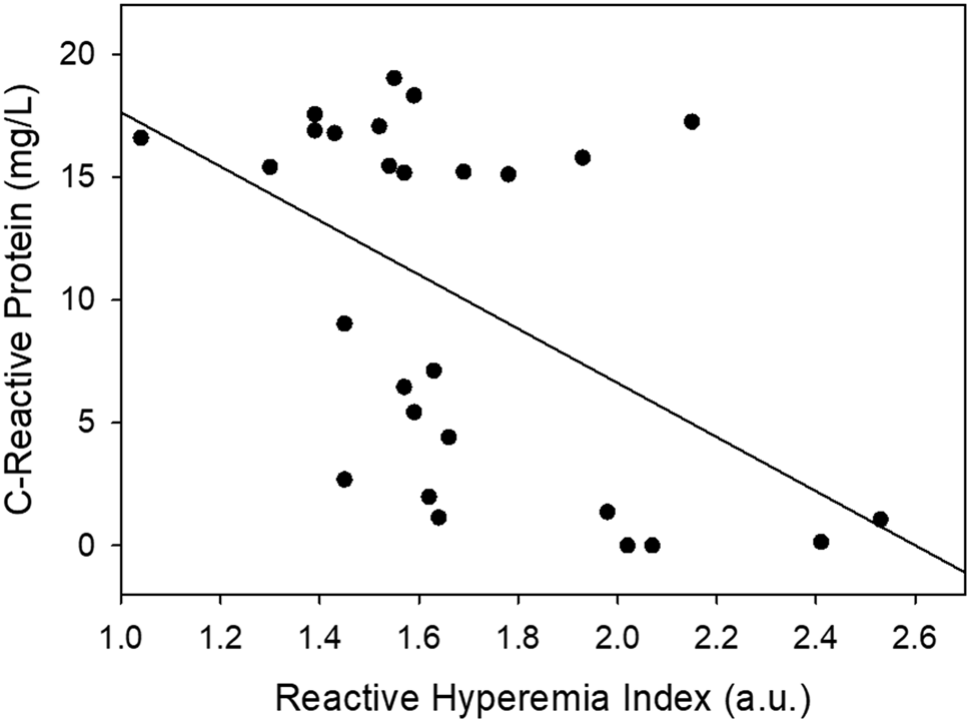
Correlation between Reactive hyperemia index (RHI) and C-Reactive Protein (CRP) at admission. (− 0.512; p = 0.008).

Measurements of RHI, PWV and CRP in stable control patients and patients with AECOPD at admission are shown in Fig. 2. The RHI was significantly lower in patients with an AECOPD compared to both GOLD 1 and 2 and GOLD 3 and 4 control COPD groups. A greater proportion of patients with an AECOPD had an impaired RHI at admission compared to control patients with stable COPD (AECOPD: 59% (31/53), GOLD 1 and 2 Stable COPD controls: 18% (2/11), GOLD 3 and 4 Stable COPD controls 33% (7/21); chi-square = 8.05; p = 0.018). Arterial stiffness was not significantly different between patients with stable COPD and patients with AECOPD at admission. Admitted patients with an AECOPD had significantly higher CRP values compared to control patients. IL-6 was not significantly different between patients with AECOPD and control patients (AECOPD inpatients: 7.2 ± 12.9; GOLD 1 and 2 Stable COPD controls: 5.8 ± 8.2; GOLD 3 and 4 Stable COPD controls: 3.2 ± 2.4; p = 0.787). A partial correlation was used to determine the relationship between RHI and CRP within the AECOPD patients while controlling for step count at admission (see Fig. 3). There was a statistically significant, moderate, negative partial correlation (− 0.512, p = 0.008) between RHI and CRP whilst controlling for step count. However, zero-order correlations showed that step count had little influence on the relationship between RHI and CRP. No relationship was present between arterial stiffness and CRP (− 0.115; p = 0.594) at admission.

**Figure 4:**
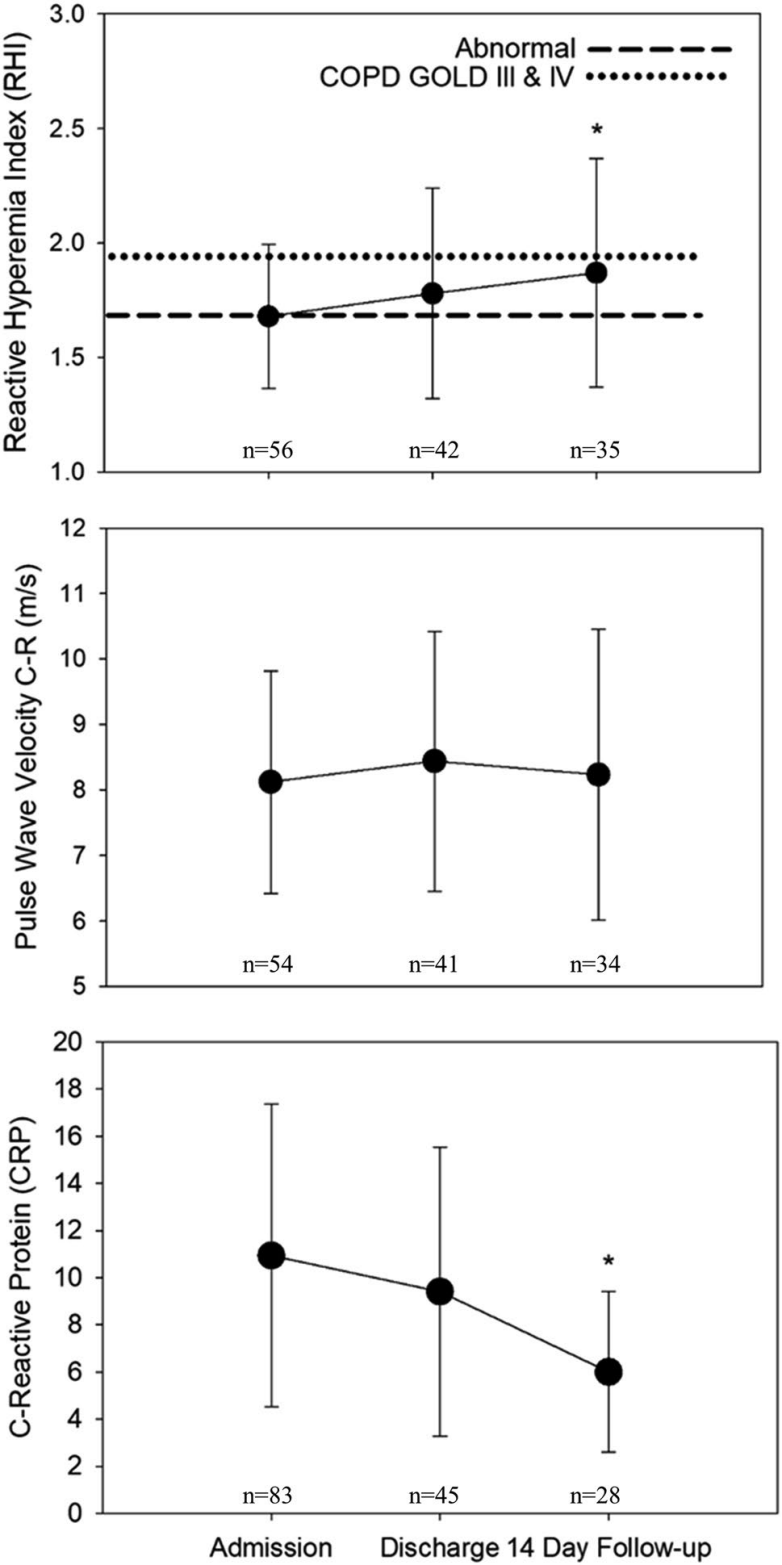
Vascular function, pulse wave velocity and systemic inflammation for patients with AECOPD across admission, discharge and 14-day follow-up. *p < 0.05 vs. admission. RHI score of < 1.67 represents endothelial dysfunction.

The time course of change in RHI, PWV and CRP are shown in Fig. 4. RHI significantly improved (estimated increase of 0.215; p = 0.031) from admission to 14 day follow up, however, regression analysis showed that age, sex, BMI and LOS did not contribute to this increase. CRP significantly decreased from admission to 14 day follow up and age, sex, FEV_1_, MAP and LOS were shown to not contribute to this difference. IL-6 and arterial stiffness remained unchanged from admission to discharge to 14 day follow-up.

### Relationship between physical activity and vascular function, pulse wave velocity, inflammation

At admission, patients with an AECOPD had significantly lower physical activity compared to controls (AECOPD: median = 830 (IQR = 1051) steps/day; GOLD 1 and 2 Stable COPD controls: 5130 (3336) steps/day; GOLD 3 and 4 Stable COPD controls: 4202 (4311) steps/day; p < 0.001). Within the AECOPD group, step count increased significantly by an estimated 1865 steps/day from admission to 14 day post discharge (p = 0.025). Age, sex, BMI and S_P_O_2_ were not statistically significant predictors, and the step count increased even after adjustment for these predictors. Step count at 14-days in patients with an AECOPD remained below the average step count in stable GOLD 1 and 2 and GOLD 3 and 4 (AECOPD 14 Day: median = 1740 (IQR = 2831) steps/day vs. GOLD 1 and 2 Stable COPD controls 5130 (3336) steps/day; GOLD 3 and 4 Stable COPD controls 4202 (4311), p = 0.02).

There was no association between step count and RHI data obtained at admission (− 0.287; p = 0.106), discharge (− 0.190; p = 0.332) and 14 day follow up (0.182; p = 0.431). Arterial Stiffness was significantly related to step count at admission (− 0.428; p = 0.012) and discharge (− 0.447; p = 0.019); however, there was no relationship at 14 day follow-up (0.071; p = 0.747). Step count and CRP showed no relationship at admission (0.111; p = 0.511), discharge (0.328; p = 0.354) or 14 day follow up (− 0.252; p = 0.454). When examining patients who experienced the greatest improvements in RHI (> 0.215), step count was not significantly different between the two RHI groups (> 0.215 units: 2450 (2302) steps/day; < 0.215 units: 4472 (4534) steps/day; p = 0.315).

## Discussion

Findings from the current study indicate that vascular function was impaired during an acute exacerbation of COPD, and this impairment was accompanied by an acute increase in systemic inflammation. Importantly, the reduction in vascular function persisted for at least 2 weeks following discharge from hospital. Surprisingly, our findings indicate that arterial stiffness was unaffected by an AECOPD. Physical activity was very low during the AECOPD hospitalization, and improved following discharge; however, physical activity did not appear to influence vascular function or inflammation in-hospital or within the first 2 weeks of discharge. The impairment in vascular function observed during an AECOPD may be an important factor contributing to the increased CV and mortality risk reported during a severe AECOPD.

Vascular function during an AECOPD hospitalization has been previously examined;^12,42^ however, our study is the first to demonstrate that vascular function is impaired when matched to patients with stable COPD. By examining vascular function at discharge and again at 2 weeks post discharge, we were able to demonstrate that while there is a relative improvement in vascular function from hospital admission, vascular dysfunction persists during recovery from an AECOPD. This provides insight into potential changes in vascular function during and following hospitalization for an AECOPD, which was previously unknown, and builds on earlier research which examined vascular function during recovery at approximately 1 month^42^ and 3 months^12^ following an AECOPD. The slight improvement in vascular function observed in the present study is consistent with previous work where FMD increased during recovery from an AECOPD hospitalization^12,42^; however and importantly, our data indicate that vascular function remains well below values observed in control patients with stable COPD. These findings suggest that persistent vascular impairment after an AECOPD hospitalization may contribute to the increased CV risk and mortality following a severe AECOPD. Recovery from an AECOPD is complex and further research targeting interventions to improve vascular health and reduce CV risk in COPD post-hospitalization is warranted.

The underlying mechanisms for impaired vascular function during an AECOPD hospitalization are not well understood. Systemic inflammation is associated with reduced vascular function in stable COPD patients^2,10^, and both Marchetti et al.^12^ and Ozben et al. (2010) postulated that the acute increase in systemic inflammation with an AECOPD likely contributed to impaired vascular function in hospitalized patients with COPD. Supporting the hypothesis of a link between systemic inflammation and impaired vascular function^2,10,12,42^, we found high circulating CRP and reduced vascular function in the AECOPD group in comparison to patients with stable COPD (see Fig. 2). Further, within the AECOPD group, a high CRP was associated with lower vascular function (see Fig. 3). Interestingly, IL-6, another marker of systemic inflammation, remained unchanged throughout the study; however, previous work has also found that CRP is elevated independently of IL-6^43^. CRP has been shown to induce vascular dysfunction by suppressing endothelial nitric oxide synthase expression and activity^44^, and our findings suggest that the acute increase in systemic inflammation observed with an AECOPD may contribute to impaired vascular function in hospitalized patients with COPD.

Surprisingly, no difference in arterial stiffness was observed between patients experiencing an exacerbation and those with stable COPD. These findings suggest that the elevated arterial stiffness often observed in patients with COPD may be the result of long-standing/chronic changes, and not impacted by a AECOPD event^45^. However these findings are contrary to previous work demonstrating an acute increase in PWV of 1.2 m/s above baseline in patients experiencing an AECOPD^8^. The divergent findings may be explained by differences in PWV assessment between the two studies. Patel et al.^8^ examined central PWV (carotid-femoral) which is predominately dependent on the elastic properties of the artery, whereas the current study assessed peripheral PWV (carotid-radial) which is predominantly dependent on the muscular properties of the artery^8^. It may be that other aspects of an AECOPD (e.g., hypoxemia, hypercapnia, medication) may influence peripheral PWV differently than central PWV and suggests that peripheral PWV may not change during a COPD hospitalization^46–48^.

As expected, physical activity was extremely low during the AECOPD hospitalization, and while step count increased following discharge, physical activity within 14 days following hospitalization was still significantly below values obtained in patients with stable COPD. Physical activity was not associated with RHI, PWV or inflammation during and following hospitalization, suggesting that physical activity/mobilization had no short-term effect on modulating CV risk during an AECOPD. One explanation for the lack of association between physical activity and CV risk parameters may be the substantially reduced physical activity observed in patients with an AECOPD throughout recovery. Average step count at 14 day follow-up in the AECOPD group remained well below the average step count observed in stable GOLD 3 and 4 patients (1740 (2831) steps/day vs. 5130 (3336) steps/day). Previous research in healthy populations has shown that increases in physical activity can reduce CV risk by as much as 21%, although this finding is based on an *increase* in step count above baseline physical activity values. In patients with stable COPD participating in pulmonary rehabilitation, the minimal clinically important difference (MCID) in step count was calculated to be between 599 to 1131 steps per day^49^. Patients who exceeded the MCID in steps per day demonstrated a significant reduction in hospital admission in the first 2 years following rehabilitation^49^. Importantly, this MCID was determined from a mean baseline of 3839 steps per day, which is well above the mean of 2730 steps per day observed 14-days following an AECOPD in the current study. It may be that comprehensive interventions such as pulmonary rehabilitation are needed to increase physical activity sufficiently to reduce CV risk following an AECOPD^50^.

As a project conducted within an acute care hospital, this study has several limitations. We were unable to assess vascular function and PWV while patients were in isolation in hospital resulting in missing data at admission and discharge (n = 12), and 22 patients were discharged before being assessed at admission. It was not possible to characterize the exacerbation into causative pathology, and medications were not standardized throughout the study. Nonetheless, most of the patients hospitalized with AECOPD received evidence-based care with systemic corticosteroids (82%) and antibiotics (79%). It was not feasible for patients to fast prior to vascular assessments, which may have impacted vascular outcomes; however, to control for this and diurnal variations, patients were assessed at the same time of day throughout the study. Because of the complexity of obtaining data within the ED, peripheral (carotid-radial) PWV data were obtained to assess arterial stiffness, which differs from central (carotid-femoral) arterial stiffness. Additionally, arterial blood gas data were not available, limiting our ability to investigate the influence of hypercapnia on PWV. At the bedside, it was not possible to perform full lung function testing, and only two flow-volume loops were conducted at admission, and therefore we were are unable to examine the association between hyperinflation and vascular function. Biomarkers of inflammation (e.g., CRP and IL-6) were selected a-priori because of their link to vascular function and PWV. We acknowledge that other inflammatory biomarkers may be altered during an AECOPD and may impact the results. Finally, control patients were recruited from our local pulmonary clinics and may not be representative of all patients with stable COPD.

An AECOPD hospitalization was associated with impaired vascular function, which improved during recovery, but remained reduced 14 days post- hospitalization relative to patients with stable COPD. Physical activity was low throughout the hospitalization, and improved following discharge; however, physical activity did not influence vascular function or inflammation. The persistent vascular dysfunction after an AECOPD hospitalization provide a mechanistic link to help explain the persistent increase in CV risk and mortality following a severe AECOPD event. In addition to focusing on respiratory recovery, clinicians would be encouraged to support the CV health of patients recovering from an AECOPD. Additional studies are needed to determine whether targeted mobilization/pulmonary rehabilitation strategies in hospital and during recovery may help normalize vascular dysfunction and CV risk in patients recovering from a COPD hospitalization.

## Data Availability

The datasets used and analyzed during the current study are available from the corresponding author on reasonable request.

## Abbreviations

AECOPD: Acute exacerbation of COPD
BNP: Brain natriuretic peptide
COPD: Chronic obstructive pulmonary disease
CAT: COPD assessment test
CRP: C-reactive protein
CV: Cardiovascular function
ED: Emergency department
FMD: Flow mediated dialatation
IL-6: Interlukin-6
IQR: Interquartile range
MAP: Mean arterial blood pressure
mMRC: Modified medical research council
PWV: Pulse wave velocity
RHI: Reactive hyperemia index
SD: Standard deviation
TnI: Troponin
